# A Case

**Published:** 1884-08

**Authors:** A. B. Knott

**Affiliations:** Monticello, Ill.


### A CASE.

A. B. Knott, M. D., Monticello, Ill.

Mrs. M., æt. fifty-seven, was taken sick September 1st, 1883. Called an allopathist, her regular attendant. She was attended by him until October 10th for colic. When I was called to the case I found the vagina prolapsed, and she complained of what seemed to be colicky pains and chills.

Chill commenced in toes, extending to the knees, and remained twelve hours. "Feet and ankles felt like as if in snow." There was cold perspiration from the beginning of the chill during the twelve hours.

The chill began at 4 A. M. and lasted till 4 P. M., and she was thirstless all the time. A peculiar symptom always present—she must lift the clothing from the abdomen (Lach.), and she would fan the abdomen with the bed-clothing. Even the vulva was hot, and she applied cold water, which relieved. Chilliness when lifting the covers and sweating only of covered parts. Smothering spells and wants to be fanned. There was no fever. The pulse was generally regular, but sometimes during the chill was feeble.

The tongue was red and appeared as if scalded. Pain in left side of head. Knees always cold. Restless sleep from 1 A. M. to 5. Pain in back when attempting to sit up. Persistent constipation. Dreams of falling, and a considerable prostration.

Some three months later, after the failure of numerous selections, I mentioned the symptoms to Dr. Ad. Lippe, while on my visit to Philadelphia. Dr. Lippe advised me to give her Ferrum. I telegraphed home to my son, Dr. J. D. Knott, to give Mrs. M., Ferr., one dose. The highest at hand was 200, which was given. She never had another chill.

I would like to have Dr. Lippe comment on this case, as there are several Hahnemannians who know of the case and would be pleased to read his able comments.

Note.—There were present in this case some characteristic symptoms belonging to the individual and not necessarily to the form of disease or to the pathological condition. First, "prolapsus vaginæ" (Ferr., Merc., Lip., Stann.); second, chill at 4 A. M. only under Ferrum; and as Ferr. is also an antidote to Chininum sulph., and whereas all the "Regulars" always administer Chininum sulph. whenever the word chill touches their tympanum, and whereas these progressive scientific medical men consider chill and malaria synonyms, and as to all appearances the chills had partaken of the characteristic symptoms belonging to Chininum sulph., Ferrum was suggested. Ad. Lippe.

